# Supplementary material for: Isoginkgetin derivative IP2 enhances the adaptive immune response against tumor antigens
Source: Commun Biol. 2021 Mar 1;4:269. doi: 10.1038/s42003-021-01801-2 (PMC7921396; doi:10.1038/s42003-021-01801-2)

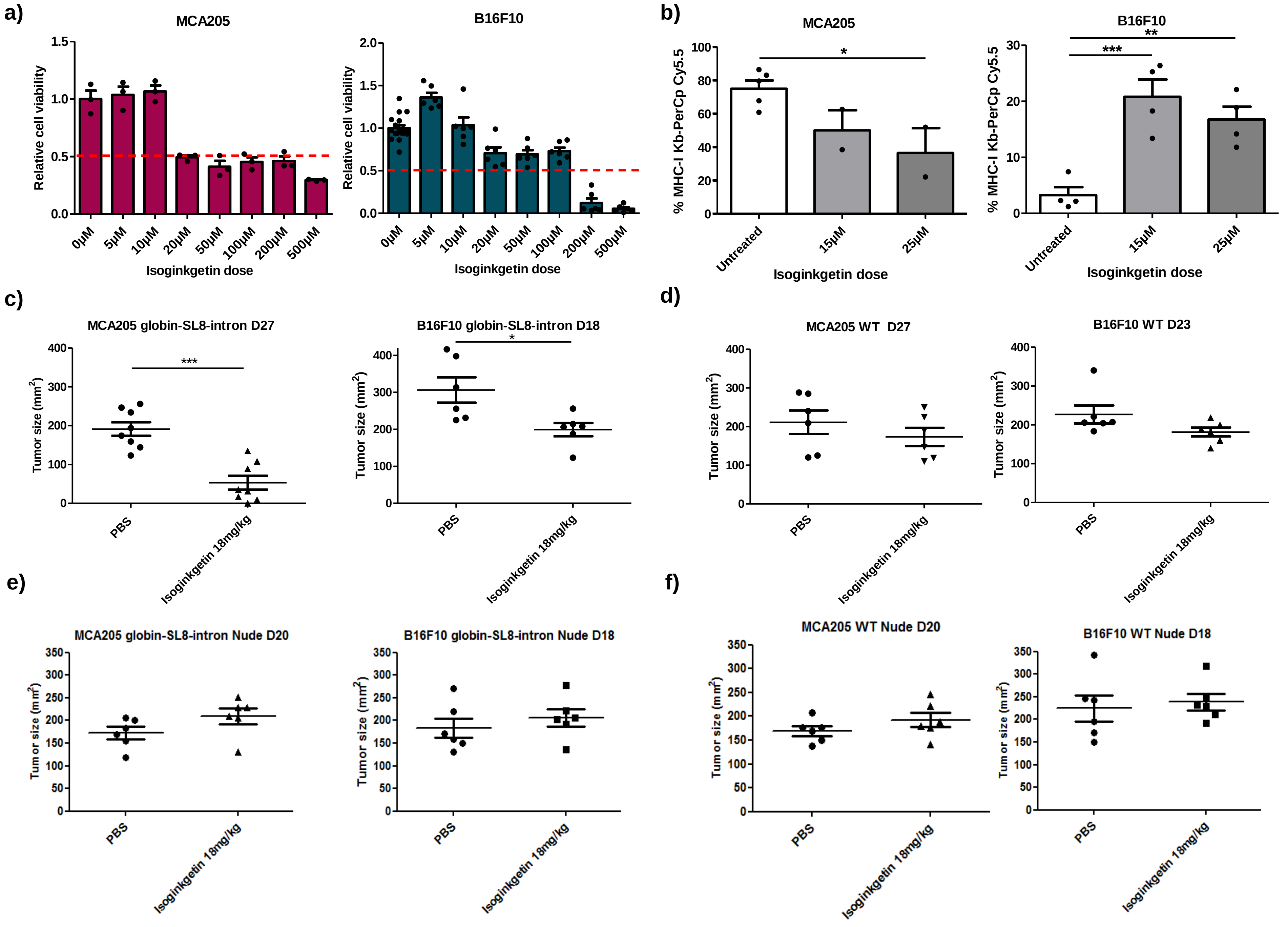

a)
MCA205
B16F10
Relative cell viability
0.0
0.5
1.0
1.5
2.0
0µM
5µM
10µM
20µM
50µM
100µM
200µM
500µM
Isoginkgetin dose
b)
MCA205
B16F10
% MHC-I Kb-PerCp Cy5.5
0
20
40
60
80
100
10
30
*
**
***
Untreated
15µM
25µM
Isoginkgetin dose
c)
MCA205 globin-SL8-intron D27
B16F10 globin-SL8-intron D18
Tumor size (mm2)
0
100
200
300
400
***
*
PBS
Isoginkgetin 18mg/kg
d)
MCA205 WT D27
B16F10 WT D23
Tumor size (mm2)
PBS
Isoginkgetin 18mg/kg
e)
MCA205 globin-SL8-intron Nude D20
B16F10 globin-SL8-intron Nude D18
Tumor size (mm2)
0
50
100
150
200
250
300
350
PBS
Isoginkgetin 18mg/kg
f)
MCA205 WT Nude D20
B16F10 WT Nude D18
Tumor size (mm2)
PBS
Isoginkgetin 18mg/kg

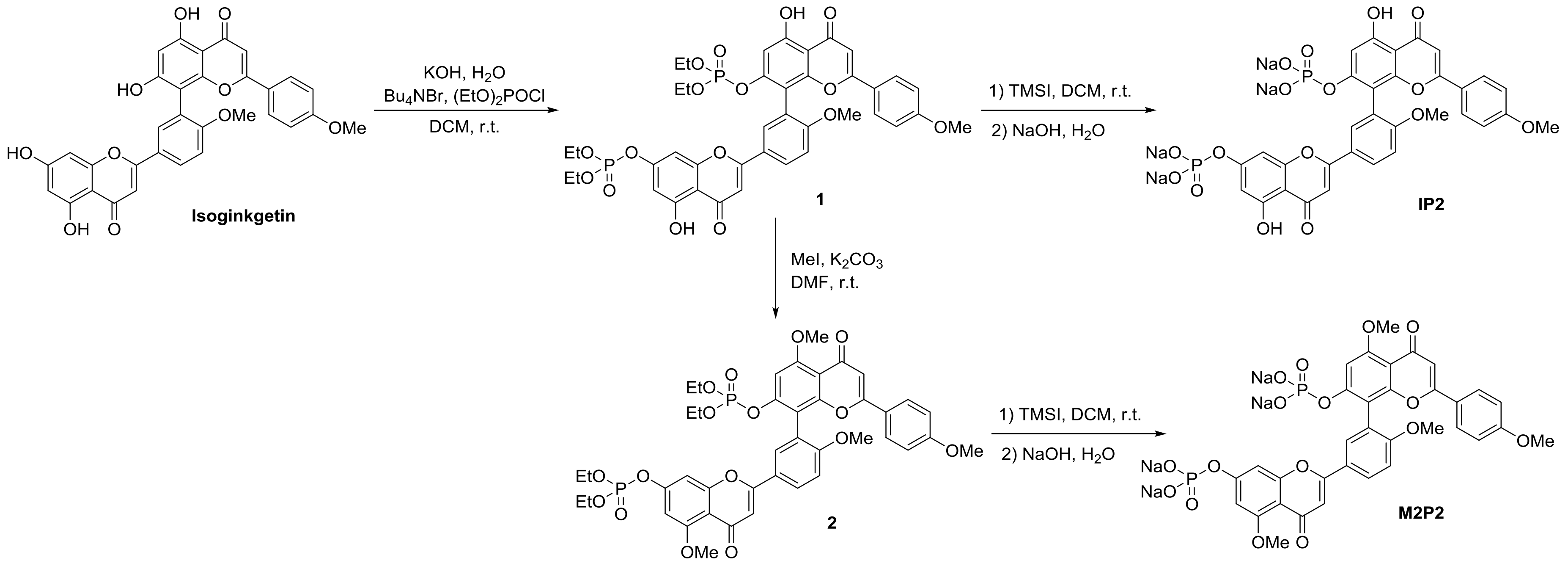
KOH, H_2O
Bu_4NBr, $(EtO)_2POCl$
DCM, r.t.
Isoginkgetin
1
1) TMSI, DCM, r.t.
2) NaOH, H_2O
IP2
MeI, K_2CO_3
DMF, r.t.
2
1) TMSI, DCM, r.t.
2) NaOH, H_2O
M2P2

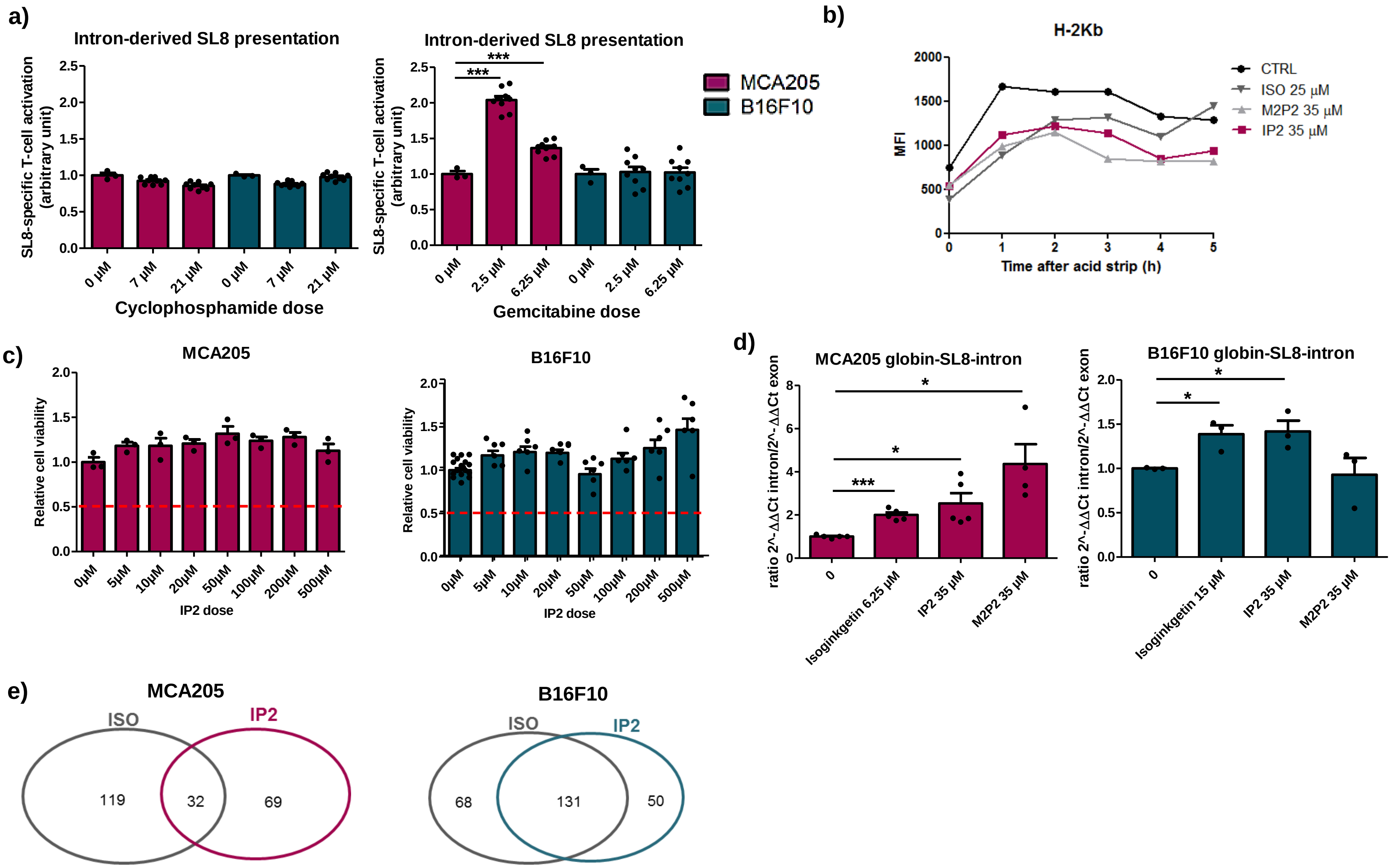
a)
Intron-derived SL8 presentation
SL8-specific T-cell activation (arbitrary unit)
0 µM
7 µM
21 µM
Cyclophosphamide dose
Intron-derived SL8 presentation
***
***
2.5 µM
6.25 µM
Gemcitabine dose
MCA205
B16F10
b)
H-2Kb
MFI
Time after acid strip (h)
CTRL
ISO 25 µM
M2P2 35 µM
IP2 35 µM
c)
MCA205
B16F10
Relative cell viability
0µM
5µM
10µM
20µM
50µM
100µM
200µM
500µM
IP2 dose
d)
MCA205 globin-SL8-intron
B16F10 globin-SL8-intron
ratio 2^-ΔΔCt intron/2^-ΔΔCt exon
Isoginkgetin 6.25 µM
Isoginkgetin 15 µM
IP2 35 µM
M2P2 35 µM
e)
MCA205
ISO
IP2
119
32
69
B16F10
68
131
50

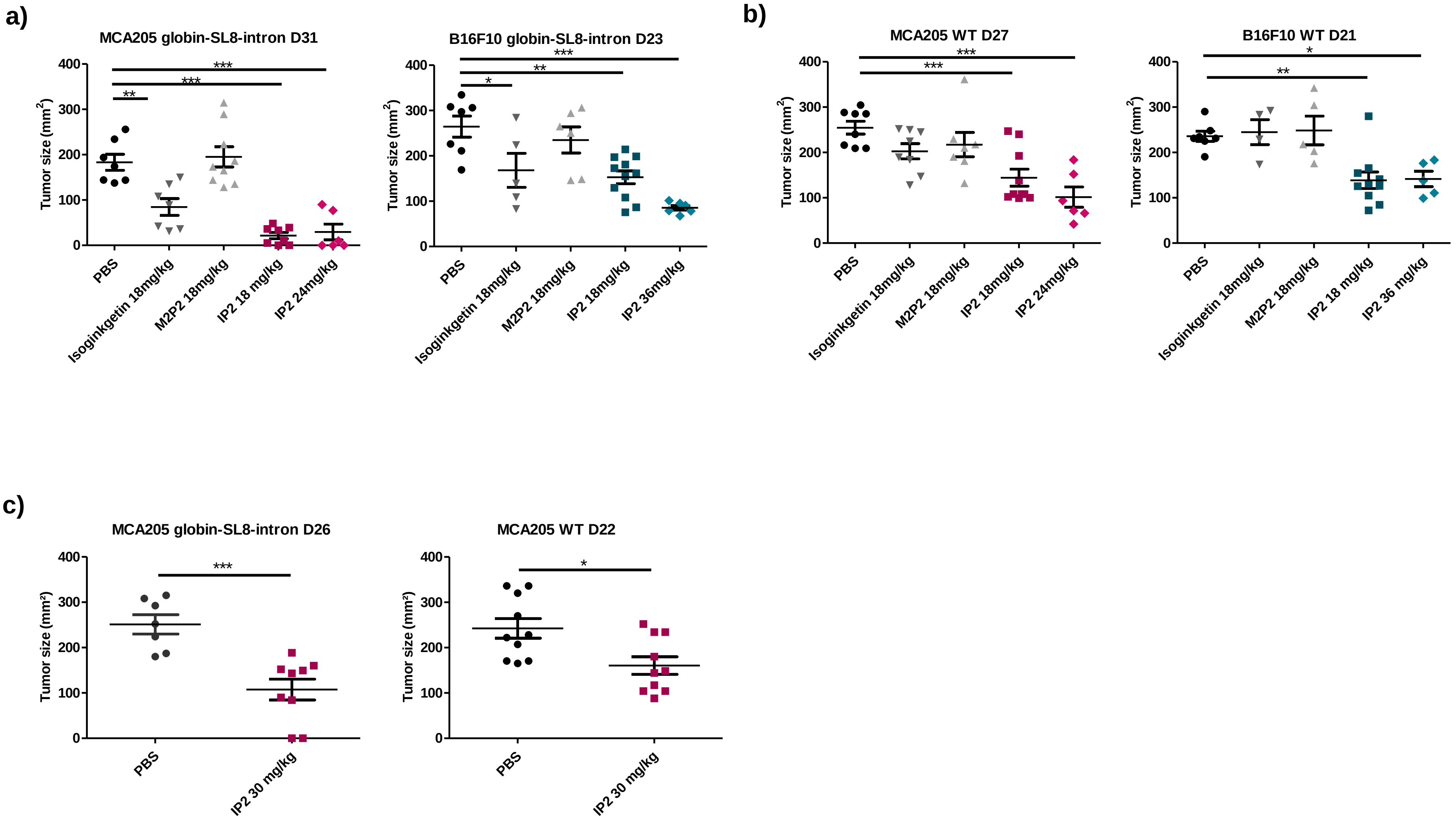
a)
MCA205 globin-SL8-intron D31
Tumor size (mm²)
0
100
200
300
400
**
***
***
PBS
Isoginkgetin 18mg/kg
M2P2 18mg/kg
IP2 18 mg/kg
IP2 24mg/kg
B16F10 globin-SL8-intron D23
Tumor size (mm²)
*
**
***
PBS
Isoginkgetin 18mg/kg
M2P2 18mg/kg
IP2 18mg/kg
IP2 36mg/kg
b)
MCA205 WT D27
Tumor size (mm²)
***
***
PBS
Isoginkgetin 18mg/kg
M2P2 18mg/kg
IP2 18mg/kg
IP2 24mg/kg
B16F10 WT D21
Tumor size (mm²)
**
*
PBS
Isoginkgetin 18mg/kg
M2P2 18mg/kg
IP2 18 mg/kg
IP2 36 mg/kg
c)
MCA205 globin-SL8-intron D26
Tumor size (mm²)
***
PBS
IP2 30 mg/kg
MCA205 WT D22
Tumor size (mm²)
*
PBS
IP2 30 mg/kg

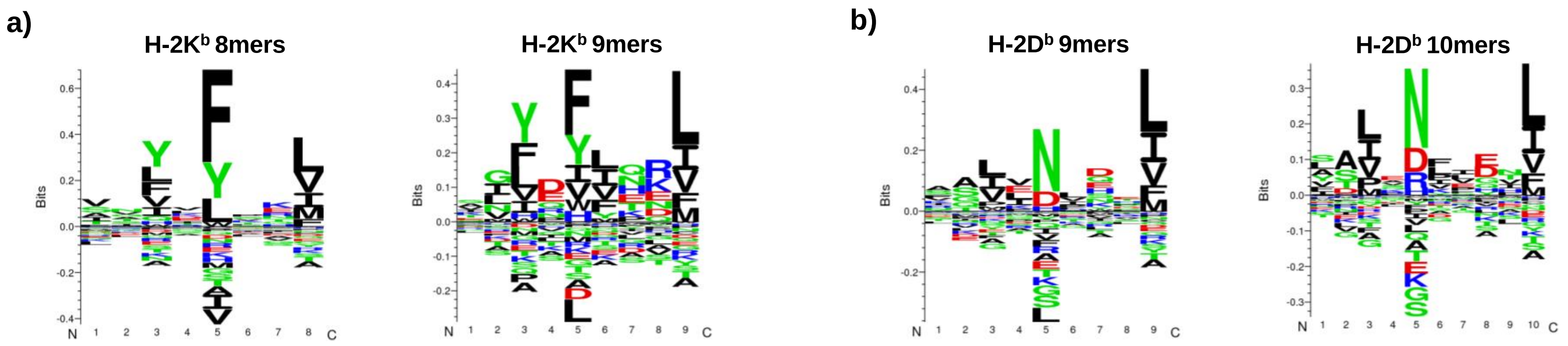
a)
H-2K^b 8mers
H-2K^b 9mers
b)
H-2D^b 9mers
H-2D^b 10mers
Bits

a)

TL9 SLP: GQIIFLPFTCIGYTDTNQDFIQRLSTLIRQAIERQLPAW

Irrelevant SLP : SIDSVRRLEHKLAEEESFPGFVNLNSTETQTAGVIDRWA

b)

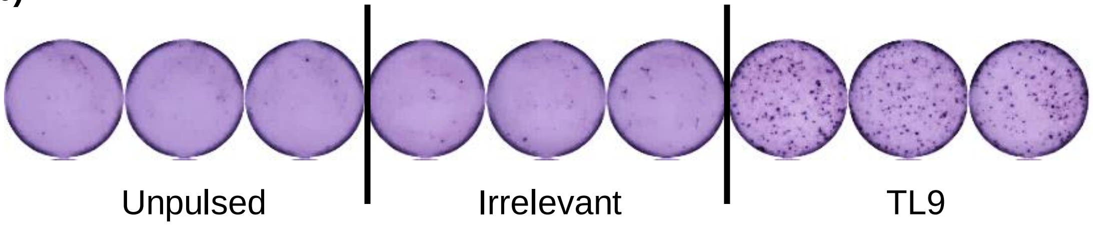

c)

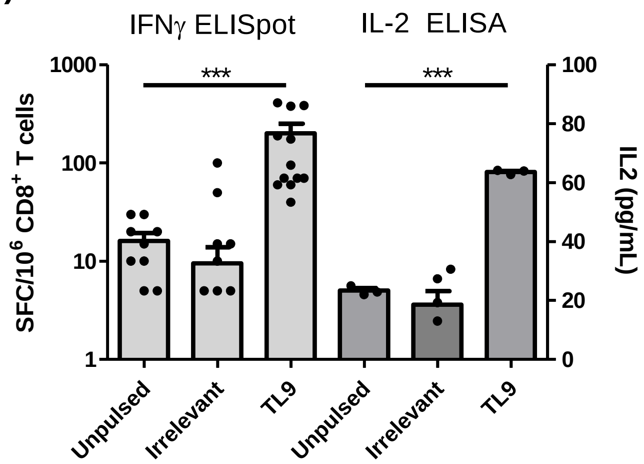

Supplement: Supplementary file 1 — Supplementary Information [file 42003_2021_1801_MOESM1_ESM.pdf]
