# Supplementary material for: Isoginkgetin derivative IP2 enhances the adaptive immune response against tumor antigens
Source: Commun Biol. 2021 Mar 1;4:269. doi: 10.1038/s42003-021-01801-2 (PMC7921396; doi:10.1038/s42003-021-01801-2)
Supplement: Supplementary file 6 — Description of Additional Supplementary Files [file 42003_2021_1801_MOESM6_ESM.pdf]

Description of Additional Supplementary Files

File Name: Supplementary Data 1

Description: List of 8-12 amino acid-long peptides identified in H-2Kb molecules at the surface of untreated MCA205 fibrosarcoma. The sequence, the length, the affinity for H-2Kb molecules computed on the NetMHC4.0 Server and the protein is provided for each epitope.

File Name: Supplementary Data 2

Description: List of 8-12 amino acid-long peptides identified in H-2Kb molecules at the surface of MCA205 fibrosarcoma treated with IP2. The sequence, the length, the affinity for H-2Kb molecules computed on the NetMHC4.0 Server and the protein is provided for each epitope. Epitopes highlighted in red were absent from untreated cells and present in at least two out of three IP2-treated replicates.

File Name: Supplementary Data 3

Description: List of 8-12 amino acid-long peptides identified in H-2Db molecules at the surface of untreated MCA205 fibrosarcoma. The sequence, the length, the affinity for H-2Db molecules computed on the NetMHC4.0 Server and the protein is provided for each epitope.

File Name: Supplementary Data 4

Description: List of 8-12 amino acid-long peptides identified in H-2Db molecules at the surface of MCA205 fibrosarcoma treated with IP2. The sequence, the length, the affinity for H-2Db molecules computed on the NetMHC4.0 Server and the protein is provided for each epitope. Epitopes highlighted in red were absent from untreated cells and present in at least two out of three IP2-treated replicates.
