# Supplementary material for: Isoginkgetin derivative IP2 enhances the adaptive immune response against tumor antigens
Source: Commun Biol. 2021 Mar 1;4:269. doi: 10.1038/s42003-021-01801-2 (PMC7921396; doi:10.1038/s42003-021-01801-2)
Supplement: Supplementary file 7 — Reporting Summary [file 42003_2021_1801_MOESM7_ESM.pdf]

nature research

Corresponding author(s): Apcher Sébastien

Last updated by author(s): Jan 21, 2021

Reporting Summary

Nature Research wishes to improve the reproducibility of the work that we publish. This form provides structure for consistency and transparency in reporting. For further information on Nature Research policies, see our Editorial Policies and the Editorial Policy Checklist.

Statistics

For all statistical analyses, confirm that the following items are present in the figure legend, table legend, main text, or Methods section.

n/a	Confirmed	
☐	☒	The exact sample size (*n*) for each experimental group/condition, given as a discrete number and unit of measurement
☐	☒	A statement on whether measurements were taken from distinct samples or whether the same sample was measured repeatedly
☐	☒	The statistical test(s) used AND whether they are one- or two-sided<br>*Only common tests should be described solely by name; describe more complex techniques in the Methods section.*
☒	☐	A description of all covariates tested
☒	☐	A description of any assumptions or corrections, such as tests of normality and adjustment for multiple comparisons
☒	☐	A full description of the statistical parameters including central tendency (e.g. means) or other basic estimates (e.g. regression coefficient) AND variation (e.g. standard deviation) or associated estimates of uncertainty (e.g. confidence intervals)
☒	☐	For null hypothesis testing, the test statistic (e.g. *F*, *t*, *r*) with confidence intervals, effect sizes, degrees of freedom and *P* value noted<br>*Give P values as exact values whenever suitable.*
☒	☐	For Bayesian analysis, information on the choice of priors and Markov chain Monte Carlo settings
☒	☐	For hierarchical and complex designs, identification of the appropriate level for tests and full reporting of outcomes
☒	☐	Estimates of effect sizes (e.g. Cohen's *d*, Pearson's *r*), indicating how they were calculated

Our web collection on statistics for biologists contains articles on many of the points above.

Software and code

Policy information about availability of computer code

Data collection	*Provide a description of all commercial, open source and custom code used to collect the data in this study, specifying the version used OR state that no software was used.*
Data analysis	*Provide a description of all commercial, open source and custom code used to analyse the data in this study, specifying the version used OR state that no software was used.*

For manuscripts utilizing custom algorithms or software that are central to the research but not yet described in published literature, software must be made available to editors and reviewers. We strongly encourage code deposition in a community repository (e.g. GitHub). See the Nature Research guidelines for submitting code & software for further information.

Data

Policy information about availability of data

All manuscripts must include a data availability statement. This statement should provide the following information, where applicable:
- Accession codes, unique identifiers, or web links for publicly available datasets
- A list of figures that have associated raw data
- A description of any restrictions on data availability

The proteomics data sets are available in the PRIDE partner repository under the identification number: PXD023019 as .raw files, .dat files and Proline 2.0 label-free quantification spreadsheet at peptide level. The Source data for graphs and charts in the main figures are available from the following repository: Dryad; Source data of main figures, Dryad, Dataset https://doi.org/10.5061/dryad.0rxwdbrzb69. All other data that support the findings of this study are available from the corresponding author upon reasonable request.

Field-specific reporting

Please select the one below that is the best fit for your research. If you are not sure, read the appropriate sections before making your selection.

- [x] Life sciences
- [ ] Behavioural & social sciences
- [ ] Ecological, evolutionary & environmental sciences

For a reference copy of the document with all sections, see nature.com/documents/nr-reporting-summary-flat.pdf

Life sciences study design

All studies must disclose on these points even when the disclosure is negative.

Sample size	no sample-size calculation was performed
Data exclusions	data were excluded from analysis when they were judged biologically incoherent or abnormally different from other replicates
Replication	in vitro experiments were performed at least three times. Technical replicates were included for each experiment. In vivo experiments were performed at least twice on a minimum of 6 mice
Randomization	Mice were allocated into experimental groups based on their tumor size prior to treatment. Experimental groups have the same mean tumor size when the first treatment is injected. Tumor size is homogeneous within a group.
Blinding	The same investigator performed randomization, data collection and analysis.

Reporting for specific materials, systems and methods

We require information from authors about some types of materials, experimental systems and methods used in many studies. Here, indicate whether each material, system or method listed is relevant to your study. If you are not sure if a list item applies to your research, read the appropriate section before selecting a response.

Materials & experimental systems

n/a	Involved in the study	
[ ]	[x]	Antibodies
[ ]	[x]	Eukaryotic cell lines
[x]	[ ]	Palaeontology and archaeology
[ ]	[x]	Animals and other organisms
[x]	[ ]	Human research participants
[x]	[ ]	Clinical data
[x]	[ ]	Dual use research of concern

Methods

n/a	Involved in the study	
[x]	[ ]	ChIP-seq
[ ]	[x]	Flow cytometry
[x]	[ ]	MRI-based neuroimaging

Antibodies

Antibodies used	Annexin V-PE (Biolegend; cat 640947; lot B260064); PE anti mouse H-2Kb (Biolegend; cat 116508, clone AF6-885; lot B238421); PerCp Cy5.5 anti mouse H-2Kb (Biolegend, cat116516; clone AF688.5); CD8a VioBright FITC (Miltenyi; 130-109-477; REA601; lot 5190924578); OVA dextramer (Immudex; JD2163; RUO; lot 20190429-EA18)
Validation	*Describe the validation of each primary antibody for the species and application, noting any validation statements on the manufacturer's website, relevant citations, antibody profiles in online databases, or data provided in the manuscript.*

Eukaryotic cell lines

Policy information about cell lines

Cell line source(s)	MCA205 were purchase from Merck and B16F10 from ATCC
Authentication	The cell lines were authenticated by Merck and ATCC
Mycoplasma contamination	All cell lines were tested for mycoplasma contamination and data are shown for mycoplasma-free cells
Commonly misidentified lines (See ICLAC register)	*Name any commonly misidentified cell lines used in the study and provide a rationale for their use.*

Animals and other organisms

Policy information about studies involving animals; ARRIVE guidelines recommended for reporting animal research

Laboratory animals	Female wild-type C57BL/6 mice at the age of 6 weeks were obtained from Harlan France. NU/NU nude mice are obtained from Charles River France
Wild animals	the study did not involve wild animals
Field-collected samples	the study did not involve field-collected samples
Ethics oversight	Animal experiments were conducted in compliance with the EU Directive 63/2010, and protocols 2016_064_5677 and were approved by the Ethical Committee of the Gustave Roussy Campus Cancer (CEEA IRCIV/IGR no. 26, registered at the French Ministry of Research)

Note that full information on the approval of the study protocol must also be provided in the manuscript.

Flow Cytometry

Plots

Confirm that:

- [x] The axis labels state the marker and fluorochrome used (e.g. CD4-FITC).
- [x] The axis scales are clearly visible. Include numbers along axes only for bottom left plot of group (a 'group' is an analysis of identical markers).
- [x] All plots are contour plots with outliers or pseudocolor plots.
- [x] A numerical value for number of cells or percentage (with statistics) is provided.

Methodology

Sample preparation	Cells are washed in PBS prior to staining. 0.5x10^6 to 1.0x10^6 cells are stained with 1 µg fluorochrome conjugated antibodies for 25min at 4°C. Prior to every staining, we used FcR Blocking Reagent (Miltenyi). After staining, cells are washed twice in PBS 0.5% BSA, 2mM EDTA
Instrument	BD LSRII
Software	FlowJo_v10
Cell population abundance	*Describe the abundance of the relevant cell populations within post-sort fractions, providing details on the purity of the samples and how it was determined.*
Gating strategy	FSC/SSC gate was used to identify cells of interest based on standard size and granularity of tumor cell lines and immune cells (lymphocytes). FSC-A/FSC-H gate was used to select single cells. Cells negative for the viability marker were further selected. Sub-populations were then identified. Boundaries of positive staining were determined using mono-stained cells for every fluorochrome

- [ ] Tick this box to confirm that a figure exemplifying the gating strategy is provided in the Supplementary Information.
